# Cyclic CMP and cyclic UMP: new (old) second messengers

**DOI:** 10.1186/1471-2210-11-S1-O34

**Published:** 2011-08-01

**Authors:** Roland Seifert, Kerstin Beste, Heike Burhenne, Ulrike Voigt, Sabine Wolter, Andreas Hammerschmidt, Daniel Reinecke, Peter Sandner, Andreas Pich, Frank Schwede, Hans G Genieser, Volkhard Kaever

**Affiliations:** 1 Institute of Pharmacology, Hannover Medical School, Germany; 2 Bayer HealthCare, Pharma Research Center, Wuppertal, Germany; 3 Institute of Toxicology, Hannover Medical School, Germany; 4 Biolog Life Science Institute, Bremen, Germany

## Historical background

As early as 1965 it was proposed that in addition to the well-established second messengers, cyclic AMP and cyclic GMP, the cyclic pyrimidine nucleotides, cyclic CMP (cCMP) and cyclic UMP (cUMP) could play a role as second messenger molecules. This hypothesis was based on the identification of cCMP- and cUMP-hydrolyzing phosphodiesterase (PDE) activities in mammalian tissues [1,2]. Additionally, extracellular effects of cCMP on cell proliferation were reported [3], but unfortunately, those effects were not reproduced. Moreover, a specific cytidylyl cyclase activity was proposed, but the methodology used was questionable [4,5]. Furthermore, cCMP and cUMP were supposedly identified by fast atom bombardment mass spectrometry, but the methodology was not sufficiently accurate for unequivocal mass determination of cCMP and cUMP [6]. Because of the substantial methodological problems, the cCMP and cUMP field had been largely abandoned. Our recent discovery that the bacterial “adenylyl” cyclase toxins, CyaA from *Bordetella pertussis* and edema factor from *Bacillus anthracis*, also exhibit substantial cytidylyl- and uridylyl cyclase activity as assessed by radiometric assays [7], renewed interest in the cCMP and cUMP field.

## Methodology

In order to critically re-evaluate the cCMP- and cUMP field, we developed highly sensitive quantitative HPLC-MS/MS mass spectrometry methods to detect multiple cyclic nucleotides (cNMPs). Unequivocal detection of cNMPs was achieved by determination of the accurate mass of the intact cNMPs and defined cNMP fragments. The MS methodology allowed us to determine cNMP levels in intact cells, organs and body fluids and to detect nucleotidyl cyclase- and PDE activities. As potential targets for cCMP and cUMP, we examined cAMP- and cGMP-activated protein kinases. For intact cell studies, we took advantage of the (*Sp*)-stereoisomers of cNMPSs and acetoxymethylesters of cNMPs. In order to identify cCMP- and cUMP-binding proteins, we used cNMP-agarose resins.

## Results and discussion

Taking advantage of the HPLC-MS/MS methodology, we detected cCMP and cUMP in numerous cultured cell types and in human urine. Growth-arrest of cells resulted in preferential decrease of cellular cCMP- and cUMP concentrations as compared to cAMP- and cGMP concentrations, indicating that cCMP- and cUMP production is dependent on factors present in serum. Known adenylyl and guanylyl cyclases exhibit, to different extents, cytidylyl- and uridylyl cyclase activities. The cNMP agarose affinity chromatography technique identified cAMP-dependent protein kinase as one target protein for cCMP and cUMP, these cNMPs being low-affinity activators of this kinase. Moreover, several recombinant cAMP- and cGMP-degrading PDEs were identified to exhibit cUMP-degrading activity, whereas to this end, we have not been able to identify a cCMP-hydrolyzing PDE. Finally, cCMP- and cUMP-acetoxymethylesters are capable of inducing differentiation of neuronal cells. In conclusion, (i) cCMP and cUMP are present at substantial concentrations in various cells types, (ii) cCMP- and cUMP concentrations are regulated by the cell proliferation status, (iii) cUMP is degraded by known PDEs, and (iv) biological effects of cCMP and cUMP are beginning to be unmasked. All these data are indicative for a role of cCMP and cUMP as (old) new second messenger molecules.
